# One Year of Experience Managing Peritonitis Secondary to Gastrointestinal Perforation at a Tertiary Care Hospital: A Retrospective Analysis

**DOI:** 10.7759/cureus.23966

**Published:** 2022-04-08

**Authors:** Muhammad Hasaan Shahid, Faisal I Khan, Zain Askri, Arslan Asad, M. Azhar Alam, Danish Ali, Rabia Saeed, Aun Jamal, Tauseef Fatima, M. Farooq Afzal

**Affiliations:** 1 General Surgery, Lahore General Hospital, Lahore, PAK; 2 General Surgery, Mansoorah Teaching Hospital, Lahore, PAK

**Keywords:** faecal peritonitis, peritonitis, duodenal perforation, ileal perforation, intestinal perforation

## Abstract

Introduction

Peritonitis secondary to gastrointestinal perforation causes high morbidity and mortality rates in the emergency department with an immediate need for surgical intervention. Despite improved surgical management procedures, patients are still suffering from gastrointestinal leak causing peritonitis that demands surgical management by highly skilled surgeons in high-quality surgical units.

Material and methods

This paper presents one year of experience in the surgical treatment of gastrointestinal perforation-related peritonitis by surgeons in Lahore General Hospital, Lahore, Pakistan. Data was retrospectively collected from patient records and quantitatively analyzed. Involved patients developed peritonitis secondary to gastrointestinal perforation requiring surgical exploration and interventions in the emergency department between November 2020 and October 2021.

Results

One hundred and fifty-eight patients were involved; the mean age was 43.46 years. The number of males was 87 (55.06%). The patients mostly presented with generalized abdominal pain (57.6%). All the patients had perforation-related peritonitis, which was most prevalent in the ileum (62%). The most performed surgical intervention was loop ileostomy (36.71%). Compared to other published reports, the incidence rate of wound dehiscence in the hospital was relatively higher. Postoperatively, wound infection was low if the skin was left open (23.62%) compared to closed skin (38.7%). Patient outcomes were acceptable as the death rate was low (3.2%, 5/158).

Conclusion

Peritonitis caused by gastrointestinal perforation is associated with a high risk of morbidity that necessitates surgical exploration. Leaving skin wound open after the surgical intervention is recommended to decrease the incidence of wound infection and dehiscence.

## Introduction

Peritonitis is an inflammatory process resulting from bacterial, fungal, and viral infections and other irritants, including granulomas and drugs [1-2]. It causes intestinal tract or free colonic perforations, which is the most frequent condition resulting in surgical emergency worldwide [3]. The perforations cause a life-threatening condition that calls for emergency surgical attention with high morbidity and mortality rates [4]. There are improvements in radiologic and surgical interventions for peritonitis, including improved intensive care management. Despite improvements and advancements in the perioperative and surgical management of fecal peritonitis perforations, the outcomes are usually not satisfactory [5-6]. Peritonitis is becoming more frequent, while optimal management procedures are still controversial. However, patients suffering from gastrointestinal perforation-related peritonitis need instant surgical management from highly skilled surgeons in a high-quality surgical unit. Surgical treatment of peritonitis perforation is a highly complex procedure, but the recent improvements in intensive care support and microbial therapy, combined with improved surgical techniques, have resulted in improved patient outcomes [4]. This paper aims to present one year of experience in the surgical management of patients presented with peritonitis secondary to gastrointestinal perforation in Lahore General Hospital, Lahore, Pakistan.

## Materials and methods

Study design

After approval from Institutional Review and Ethical Board, this retrospective cross-sectional study was carried out over one year period from November 2020 and October 2021; the patients were surgically managed in the Accident and Emergency Department of Lahore General Hospital, Pakistan.

Inclusion and exclusion criteria

All the patients with peritonitis secondary to gastrointestinal perforation were included in the study. Patient records and surgery notes were used to compile the data. The patients in the study were from 15 to 68 years old.

Patients having peritonitis due to causes other than gastrointestinal perforation, such as biliary peritonitis or spontaneous bacterial peritonitis, were excluded from the study. 

Data collection and patient management

A detailed history of the start of discomfort, anorexia, vomiting, and fever was taken. A general survey was conducted, with a focus on measuring pulse, temperature, and blood pressure. Pain on palpation, generalized tenderness, and guarding test were all part of the abdominal examination. Every patient had to undergo a rectal examination.

To control symptoms of sepsis, other systems were checked. After provisionally diagnosing the patient with peritonitis, additional tests to confirm the diagnosis included a total count to check for leucocytosis, a biochemical examination to check for blood sugar, urea, and creatinine, an upright X-ray abdomen, and an ultrasound. For all patients with peritonitis, a final decision on operational intervention was taken.

Due to technical difficulties and/or an advanced stage of infection, such as four-quadrant pus, the laparoscopy was modified to an open laparotomy (incision from xiphisternum to pubic symphysis) or lower midline laparotomy (incision from the umbilicus to pubic symphysis). Histopathological evaluation of the resected tissue was performed.

Patients with peritonitis were resuscitated with IV fluids as well as with a low dose of inotropic support if required. Intravenous broad-spectrum antibiotics were given during the preoperative period as well as during the postoperative hospital stay, including third-generation cephalosporins and meropenem. 

Analgesics were administered in the form of ketorolac injections for a period of 24 hours. Additional analgesics were prescribed depending on the patients' pain perception.

The operating time and the length of hospital stay were recorded as comparable data. The patients were encouraged to return to their regular activities and work as soon as they felt ready. Normal activity was defined as the patient's return to normal household and social activities of their choosing. For one month, the patients were followed up on weekly basis, but none of the patients required readmission.

Statistical analysis

Descriptive statistics and regression analysis were used for the analysis of data. A p-value <0.05 was considered significant. The Statistical Package for the Social Sciences (SPSS) version 24 for Windows (IBM Inc., Armonk, USA) was used to statistically evaluate the data obtained.

## Results

Demographics

The number of patients that underwent laparotomies in the Lahore General Hospital surgical unit was 158. The mean age was 34.46 years. The number of patients aged 17 years and younger was five (3.16%), 90 (56.96%) patients were aged between 18 and 34 years, 31 (19.62%) were aged between 35 and 44 years, 22 (13.92%) were aged between 45 and 65 years, 10 (6.63%) were 65 years and older. Male patients were 87 (55.06%), and females were 71 (44.947%).

Performed procedures

The surgical unit of the hospital performed three surgical approaches to explore the patients with peritonitis. That included exploratory laparotomy (incision from xiphisternum to pubic symphysis), laparoscopy converted into exploratory laparotomy (incision from xiphisternum to pubic symphysis), and lower midline laparotomy (incision from the umbilicus to pubic symphysis). The decision on the surgical approach was made perioperatively according to the patient's need (Table 1). Out of 158, 135 (85.4%) patients had exploratory laparotomy, laparoscopy was converted into exploratory laparotomy for 16 (10.3%) patients, and seven (4.43%) had lower midline laparotomy.

**Table 1:** Surgical examination procedures performed on the patients

Procedure	Frequency (n)	Percent (%)
Exploratory laparotomy (incision from xiphisternum to pubic symphysis)	135	85.44
Laparoscopy converted into exploratory laparotomy (incision from xiphisternum to pubic symphysis)	16	10.13
Lower midline laparotomy (incision from the umbilicus to pubic symphysis)	7	4.43
	158	100.00

Presentations

Among the 158 patients, 40 (25.3%) were in moderate to severe septic shock at presentation, while 118 (74.68) were vitally stable or in mild shock. During the preoperative assessment, the patients presented with generalized abdominal pain, epigastric pain, constipation, and fever. More than half of the patients (91, 57.6%) presented with generalized abdominal pain, 84 (53.1%) of them presented with constipation, 48 (30.3%) of them presented with fever, 23 (14.56%) presented with epigastric pain (Table 2).

**Table 2:** Preoperative presentations of the patients

Findings	Frequency (n)	Percentage (%)
Generalized abdomen pain	91	57.59
Epigastric pain	23	14.56
Constipation	84	53.1
Fever	48	30.3

The findings from the surgical examination include the presence of perforation, the site, and the size of the perforations.

Etiology

Out of 158 patients, 28 (17.7%) presented with peritonitis due to gastrointestinal perforation and had a history of nonsteroidal anti-inflammatory drugs (NSAIDs) intake, while in four cases, *H. pylori* was isolated. Thirty-two patients had peritonitis due to gastric or duodenal perforation. Thirty-six (22.7%) patients had a history of smoking. Out of 112 patients having Ileal perforations, cultures showed the presence of *Salmonella typhi *in 84 patients, while 26 patients developed Ileal perforation due to abdominal tuberculosis. Three patients had a history of instrumentation leading to colonic perforation. The rest of the patients had a non-specific type of inflammation leading to intestinal perforation.

Perforation

As all the patients had peritonitis due to GI perforations, more than two-thirds (71.3%) of the patients had perforations in their ileum, 14 (8.86%) had it in the stomach, three (1.9%) had a perforation in the jejunum, 21 (13.29) patients had the perforation in the duodenum, and eight (5.06%) of them had it in the colon (Table 3). 

**Table 3:** Site of perforation

Site	Frequency (n)	Percentage (%)
Jejunum	3	1.90
Duodenum	21	13.29
Stomach	14	8.86
Ileum	112	71.30
Colon	8	5.06
	158	100.00

Perforation size

Regarding the size of the perforations, 41 (26%) had pinpoint to 0.25 cm^2,^ more than half (87, 55%) of the patients had the perforation size of >0.25 to 1.0 cm^2,^ 10 (6.3%) had >1 cm^2^ to 2.25 cm^2,^ 19 (12%) had >2.25 to 4 cm^2^, and only one patient (0.6%) had perforation size of 9 cm^2^ (Table 4).

**Table 4:** Size of perforation

Size (cm^2^)	Frequency (n)	Percentage (%)
Pinpoint to 0.25	41	25.95
>0.25 to 1	87	55.06
>1 to 2.25	10	6.33
>2.25 to 4	19	12.03
>4	1	0.63
	158	100.00

Intervention

Nine interventions were performed for the patients. The interventions are primary repair, loop ileostomy, resection and double barrel ileostomy, resection and double barrel colostomy, primary repair and diversion ileostomy, primary repair and diversion colostomy, Garahm omentopexy, modified Garahm omentopexy and ileocolic anastomosis, and hemicolectomy with ileostomy and mucous fistula. The number and percentage of each of the interventions performed are shown in Table 5.

**Table 5:** Patients receiving the interventions

Intervention	Frequency (n)	Percentage (%)
Primary repair	3	1.90
Loop ileostomy	58	36.71
Resection and double barrel Ileostomy	40	25.32
Resection and double barrel colostomy	5	3.16
Primary repair and diversion ileostomy	14	8.86
Primary repair and diversion colostomy	4	2.53
Garahm omentopexy	4	2.53
Modified Garahm omentopexy	17	10.76
Hemicolectomy with ileostomy and mucous fistula	13	8.23
	158	100.00

Fascial dehiscence

Only 20 (12.7%) patients had fascial dehiscence. Among them, the skin wound of 11 patients was left open for secondary or tertiary healing, while the skin wound of nine patients was closed primarily (Table 6). The regression analysis revealed a statistically significant difference between the skin closure and fascial dehiscence (p<0.007).

**Table 6:** Skin wound and fascial dehiscence

Skin	Fascial dehiscence			
	Yes (n)	Percentage (%)	No (n)	Percentage (%)
Left open	11	6.9%	115	72.7%
Closed primarily	9	5.7%	23	14.5%
Total	20	12.6%	138	87.3%

Skin 

The skin was either left open for secondary or tertiary healing or closed after the surgical intervention. For 127 (80.3%) of the patients, the skin was left open, while other patients (31, 19.7%) had their skin closed after the surgical interventions. Forty-two patients had a superficial surgical site infection. Thirty patients with their skin left open after the surgery had an infection, while 12 patients with their skin closed had an infection (Table 7). The result of the regression analysis revealed a statistically not significant difference between skin left open or closed and wound infection (p=0.072).

**Table 7:** Skin (closed or left open) and wound infection

Skin	Wound infection			
	Yes (n)	Percentage (%)	No (n)	Percentage (%)
Left open	30	23.62	97	76.37
Closed	12	38.7	19	61.2
	42		116	

Patient outcomes

One hundred and fifty-four patients were discharged with follow-up. Four patients died after the intervention due to advanced sepsis. Among them, three patients had an ileostomy, and one patient had modified Garahm omentopexy.

## Discussion

The spectrum of fecal peritonitis cases in Pakistan is different from developed countries. It is different regarding etiological factors, younger age at presentation, and site of perforation. As in our study, upper GI was the most commonly involved part compared to western and far east countries, where lower GI tracks are usually the most common areas of perforation [7].

Most of the patients were young adults aged between 18 and 34 years. That can be attributed to unhealthy eating habits among the population [8]. In our study, 28.3% of patients were presented with moderate to severe shock, and the most common patient presentation was generalized abdominal pain which was supported by results shown in a study done in Sindh, Pakistan, by Memon et al. where abdominal tenderness was present in 85% of patient and 83% of the patients had rigidity in their abdomen [9-10]. Peritonitis remains a significant cause of morbidity and mortality on the emergency floor. Patients with peritonitis usually undergo emergency surgery [11]. In our study, out of 158 patients presenting in ER with peritonitis, 135 (85.4%) patients underwent exploratory laparotomy, for 16 (10.3%) patients, diagnostic laparoscopy was converted into exploratory laparotomy (incision from xiphisternum to pubic symphysis) and a lower midline laparotomy (incision from xiphisternum to pubic symphysis) was performed in seven (4.43%) patients during the study period. In our study, more than two-thirds of the patients had a perforation in the ileum, while stomach and jejunum perforation had the second most common incidence. Findings from other studies done in the southern region of Asia also support that perforation is a common occurrence in patients with peritonitis [12].

Our results showed perforation in the ileum was most common in our patients (71.3%). This research indicates that peritonitis due to perforation is usually found in the ileum, which supports Wani et al., who found the occurrence of perforation in peritonitis cases [13].

The most performed intervention was loop ileostomy. Wound dehiscence recorded in the hospital was similar to findings from other studies, indicating that wound dehiscence is not a common event in the surgical treatment of peritonitis if managed properly. The regression analysis indicated that wound dehiscence could be attributed to the performed surgical intervention. Compared to findings from other studies, the rate of wound dehiscence in the hospital was relatively higher [14-15].

## Conclusions

Peritonitis caused by gastrointestinal perforation is associated with a high risk of morbidity requiring surgical exploration. Fascial dehiscence is a common occurrence, and the rate of occurrence was higher than reported in other studies. Furthermore, leaving skin wounds open after the surgical intervention is recommended to mitigate the incident rates of wound infection as well as fascial dehiscence.
